# Safety assessment of Edaravone: A real-world adverse event analysis based on the FAERS Database

**DOI:** 10.1371/journal.pone.0335362

**Published:** 2025-10-23

**Authors:** Ziyue Wang, Bowen Lu, Hao Yang, Weijie Zhao, Xinru Kong, Chuanhao Mi, Jianlin Wu

**Affiliations:** 1 College of Traditional Chinese Medicine, Shandong University of Traditional Chinese Medicine, Jinan City, Shandong Province, China; 2 Department of First Clinical Medical College, Shandong University of Traditional Chinese Medicine, Jinan City, Shandong Province, China; 3 Zhangqiu District Traditional Chinese Medicine Hospital, Jinan City, Shandong Province, China; 4 Department of Vertigo Center, Air Force Medical Center, PLA, Beijing, China; First Hospital of Jilin University, CHINA

## Abstract

**Objective:**

Edaravone is a neuroprotective agent, but the characteristics of its adverse events (AEs) remain insufficiently explored. This study aims to examine AEs associated with edaravone use by analyzing real-world data from the FDA Adverse Event Reporting System (FAERS).

**Methods:**

This retrospective study extracted adverse event reports related to edaravone from the FAERS database, spanning from the second quarter of 2017 to the second quarter of 2024. Disproportionality analysis methods, including the Reporting Odds Ratio (ROR), Proportional Reporting Ratio (PRR), and Bayesian Confidence Propagation Neural Network (BCPNN), were employed to detect AE signals associated with edaravone use.

**Results:**

Among 2,931 adverse event reports (AERs) in which edaravone was identified as the primary suspected drug, 86 preferred terms (PTs) and 20 system organ classes (SOCs) were included. At the PTs level, the significant drug-related adverse events were death (n = 589, ROR = 8.64), disease progression (n = 266, ROR = 28.26) and drug ineffectiveness (n = 252, ROR = 2.16). Additionally, rare but notably strong adverse event signals were observed, including thrombosis at the catheter site thrombosi, gastric fistula, and vein collapse.

**Conclusion:**

Our research found that edaravone has some overlooked adverse reactions. Further epidemiological studies are needed to more comprehensively explore and assess the risk-benefit profile of edaravone.

## 1. Introduction

Edaravone is a free radical scavenger with significant biological activity, including antioxidant, neuroprotective, and anti-inflammatory effects [1]. As a neuroprotective agent, edaravone works by scavenging free radicals in the body to prevent damage caused by oxidative stress [2]. Initially developed as a neuroprotective drug for acute ischemic stroke (AIS), edaravone has since been proven to effectively slow the progression of amyotrophic lateral sclerosis (ALS) and became the second drug approved for ALS treatment [3]. Edaravone was officially approved in Japan in 2001 for the treatment of AIS [4] and has been widely used in several Asian countries, including China [5] and India [6]. Furthermore, edaravone was approved for ALS treatment in Japan in 2015 and in the United States in 2017 [7].

As a scavenger of peroxyl radicals and peroxynitrite, edaravone provides cellular and neuroprotective benefits by eliminating hydroxyl radicals and lipid peroxides, thereby preventing neuronal damage due to oxidative stress [8]. By neutralizing reactive oxygen species (ROS), edaravone prevents neurodegeneration and motor neuron death [9]. Additionally, edaravone helps maintain cerebral blood flow by reducing oxidative stress, thereby exerting its neuroprotective effect [10]. Although edaravone is known to reduce inflammation, restore neuronal structure, and provide neuroprotection, studies have shown that its use is associated with an adverse events (AE) incidence rate as high as 84%. Common adverse reactions include bruising, constipation, and upper respiratory tract inflammation [11]. Increasing evidence suggests that edaravone is effective in treating various diseases associated with oxidative stress. However, like all pharmacological treatments, the use of edaravone carries the potential for adverse reactions. Therefore, the development of safe and effective drug treatments remains crucial [3]. In the real world, comprehensive reports on the AEs associated with edaravone are lacking. Thus, it is essential to employ data mining techniques to conduct a safety analysis of edaravone.

The FDA Adverse Event Reporting System (FAERS) is a database that collects and analyzes AEs related to drug use. It stores a vast number of reports on drug-related AEs and provides a rich data source for evaluating drug safety and efficacy [12]. This study aims to collect and analyze data on adverse reactions associated with edaravone through the FAERS database to elucidate the safety profile of edaravone, optimize treatment regimens, reduce adverse reactions, and guide clinicians in the rational and standardized use of the drug, ultimately ensuring patient safety.

## 2. Materials and methods

**Fig 1:**
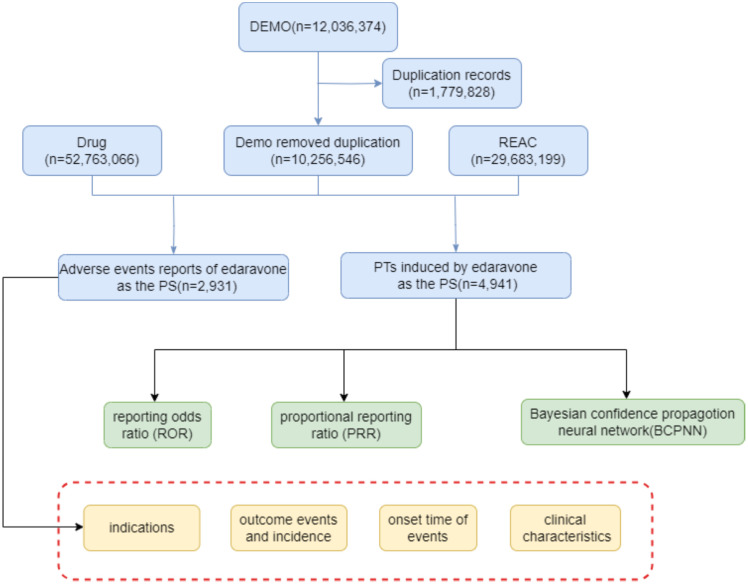
The flow diagram of selecting edaravone-related AEs from FAERS database.

To determine adverse drug reactions linked to edaravone, this study analyzed data from the FAERS database using three statistical algorithms. The workflow of the method (Fig 1).

### 2.1. Data source

This study utilizes the FAERS database, which is primarily used for the safety monitoring of drugs and biologics [13]. The FAERS database is updated quarterly and includes individual safety reports, drug information, adverse events, outcomes, report sources, treatment duration, and indications [14]. Data from the second quarter of 2017 to the second quarter of 2024 were extracted for this study. The data were processed using R statistical software (version 4.4.1).

### 2.2. Data extraction and analysis

To ensure the accuracy of the data, a preprocessing step was performed on the FAERS data to identify and remove duplicate reports, thereby standardizing the dataset. Specifically, for reports with the same CASEID (Case ID, the unique identification code for each adverse event), the report with the most recent FDA_DT (Report Date, the date when the report was entered into the database) was retained. When both CASEID and FDA_DT were identical, the report with the higher PRIMARYID (Report ID, the unique identifier automatically generated by the system) was kept [15,16]. Next,the Medex_UIMA natural language processing system (version 1.8.3) was used to standardize non-standardized drug names and to classify reports related to adverse events associated with edaravone use [17].

Additionally, three methods were used to detect AE signals, including the Reporting Odds Ratio (ROR), Proportional Reporting Ratio (PRR), and Bayesian Confidence Propagation Neural Network (BCPNN) [18]. ROR is a traditional signal detection method that corrects for biases caused by low report frequencies, helping to identify potential associations between a drug and an AE [19]. Compared to ROR, PRR is more specific. BCPNN is particularly effective in detecting unique signals, even when there are fewer reports of AEs [20]. Chi-square tests were used to compare proportions, and a ranking scale was applied to prioritize signals [21]. A combination of algorithms was used in this study to leverage their respective advantages, broaden the detection scope, and validate the results from multiple perspectives. By integrating these methods, cross-validation can be performed to minimize false positives, and adjustments to thresholds and variance allow for the detection of more potential rare AEs. All calculations were based on the 2 × 2 tables(S1 Table), with specific formulas and thresholds provided in S2 Table. Statistical analysis was performed using Microsoft Excel 2021 [22].

### 2.3. Signal filtering and classification

Preferred Terms (PTs, used to describe adverse events or medical procedures) with a report count ≥3 were chosen. The signals were then encoded, classified, and located using the International Medical Dictionary (MedDRA) PT and System Organ Class (SOC,one of the classification systems used in MedDRA for grouping diseases and adverse events) dictionaries,facilitating the analysis of specific SOCs related to the AE signals [19,23].

### 2.4. Ethics statements

All data used in this study were secondary data obtained from public databases. Ethical approval was not required.

## 3. Results

### 3.1. Basic information on AEs related to edaravone

**Fig 2:**
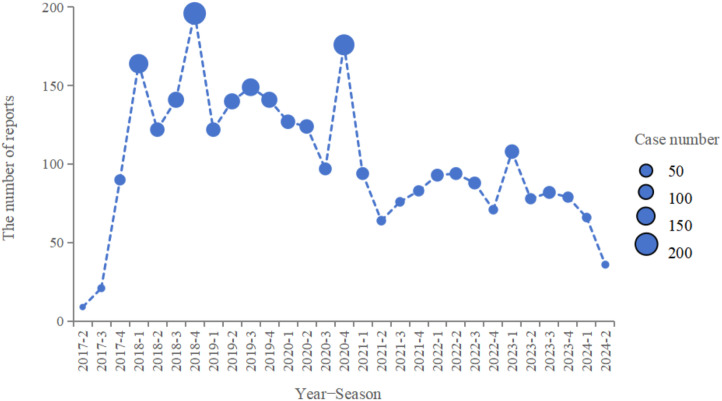
Distribution of AEs edaravone from the second quarter of 2017 (2017 Q2) to the second quarter of 2024 (2024 Q2).

From Q2 2017 to Q2 2024, a total of 2,931 reports identified edaravone as the primary suspect drug for AEs (Table 1). The annual distribution of AE reports related to edaravone is shown in Fig 2. The top three quarters with the highest number of reports were Q4 2018 (196 reports), Q4 2020 (176 reports), and Q1 2018 (164 reports). ALS was the most frequently reported indication (n = 2,025, 72.45%), followed by products used for unspecified indications (n = 675, 24.15%) and cerebral infarction (n = 58, 2.08%) (Table 2).

**Table 1:** Basic information on AEs related to Edaravone from the FAERS database.

Variable	Total
**Sex**	
Female	796 (27.16)
Male	1193 (40.70)
Unknown	942 (32.14)
**Age_yrq**	
<18	8 (0.27)
18 ~ 45	35 (1.19)
45 ~ 60	208 (7.10)
>=60	663 (22.62)
Unknown	2017 (68.82)
**wt**	70.00 (57.00,81.27)
**Reporter**	
Consumer	1724 (58.82)
Physician	647 (22.07)
Pharmacist	285 (9.72)
Other health-professional	271 (9.25)
Unknown	3 (0.10)
Lawyer	1 (0.03)
**Reported countries**	
United States	2287 (78.03)
Japan	461 (15.73)
Other	183 (6.24)
**Route**	
Other	1274 (43.47)
Intravenous	1015 (34.63)
Intravenous drip	426 (14.53)
Oral	190 (6.48)
Parenteral	26 (0.89)
**Outcomes**	
Death	962 (55.48)
Other serious	375 (21.63)
Hospitalization	362 (20.88)
Life threatening	19 (1.10)
Disability	11 (0.63)
Required intervention to Prevent Permanent Impairment/Damage	5 (0.29)
**ttoQ**	
<7	134 (10.79)
7 ~ 28	76 (6.12)
28 ~ 60	34 (2.74)
>=60	160 (12.88)
Unknown	838 (67.47)

**Table 2:** Number of indication reports for edaravone based on the FAERS database.

id	Edaravone	n(%)
1	Amyotrophic lateral sclerosis	2025 (72.45)
2	Product used for unknown indication	675 (24.15)
3	Cerebral infarction	58 (2.08)
4	Thrombotic cerebral infarction	5 (0.18)
5	Lacunar infarction	3 (0.11)

FAERS data reflect real-world drug use and adverse event reporting and do not necessarily correspond to FDA-approved indications.

In terms of gender, the number of male patients significantly exceeded that of female patients (40.70% vs. 27.16%). Regarding age, over 68.82% of the reports lacked age information, limiting a deeper analysis of the relationship between age and AEs. Among the available age data, older adults aged 60 and above were the most frequently reported group. As for the timing of onset, AEs occurring two months or more after drug administration were the most common (12.88%), followed by those within one week of use (10.79%). A total of 838 reports (67.47%) did not specify the timing of AE occurrence. Furthermore, most reports were submitted by consumers (58.82%), with 22.07% from physicians and 9.72% from pharmacists. Geographically, most reports originated from the United States (78.03%) and Japan (15.73%), collectively accounting for 93.76% of the total reports. In terms of clinical outcomes, apart from unspecified AEs, fatal outcomes were the most common (55.48%), followed by other serious outcomes and hospitalizations. Detailed information is presented in Table 1.

### 3.2. Signal detection for edaravone

**Fig 3:**
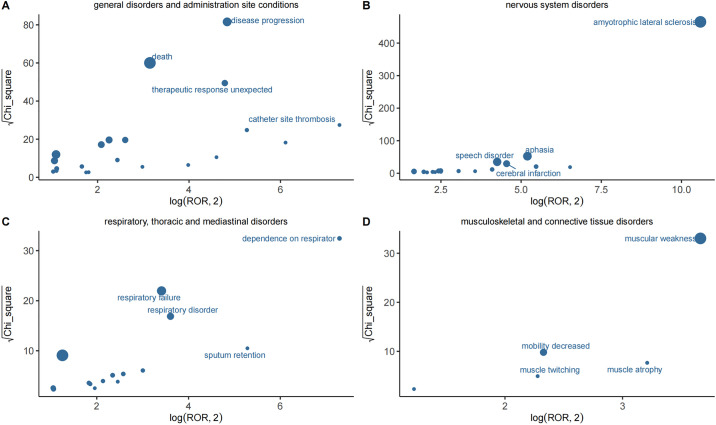
PTs distribution diagram at four common SOC levels.

The analysis of AERs associated with edaravone identified adverse reactions involving 14 SOCs. The four most notable SOCs were general disorders and administration site conditions (n = 2,140), nervous system disorders (n = 717), respiratory, thoracic, and mediastinal disorders (n = 358), and musculoskeletal and connective tissue disorders (n = 238) (Fig 3). Additionally, certain AEs, including infections and infestations (n = 242), vascular and lymphatic disorders (n = 93), and gastrointestinal disorders (n = 196), were identified as unique to edaravone (Table 3). Notably, musculoskeletal and connective tissue disorders, as well as injury, poisoning, and procedural complications, were not mentioned in the drug’s prescribing information, warranting further attention and research.

**Table 3:** The number of adverse event reports for cases at each System Organ Class (SOC) level.

SOC_English	Case Reports	IC(IC025)
General disorders and administration site conditions	2140	1.24 (1.16)
Nervous system disorders	717	0.87 (0.76)
Respiratory, thoracic and mediastinal disorders	358	0.61 (0.45)
Vascular disorders	93	−0.07 (−0.36)
Musculoskeletal and connective tissue disorders	238	−0.14 (−0.33)
Infections and infestations	242	−0.22 (−0.41)
Hepatobiliary disorders	28	−0.62 (−1.14)
Cardiac disorders	66	−0.67 (−1.02)
Metabolism and nutrition disorders	63	−0.7 (−1.06)
Investigations	160	−0.9 (−1.13)
Renal and urinary disorders	50	−1.06 (−1.45)
Injury, poisoning and procedural complications	289	−1.04 (−1.21)
Gastrointestinal disorders	196	−1.09 (−1.29)
Skin and subcutaneous tissue disorders	127	−1.25 (−1.5)
Immune system disorders	24	−1.4 (−1.97)
Psychiatric disorders	82	−1.75 (−2.06)
Blood and lymphatic system disorders	25	−1.79 (−2.34)
Ear and labyrinth disorders	6	−1.87 (−2.94)
Eye disorders	15	−2.73 (−3.44)
Neoplasms benign, malignant and unspecified (incl cysts and polyps)	15	−3.5 (−4.21)

At the PTs level, 86 potential PTs were identified through three algorithms assessing adherence to various screening criteria. Based on the ROR algorithm, PTs were ranked in descending order of signal strength (95% CI values), with ALS showing the strongest signal. The ten PTs with the highest number of cases were death (n = 589, ROR = 8.64), disease progression (n = 266, ROR = 28.26), drug ineffectiveness (n = 252, ROR = 2.16), ALS (n = 182, ROR = 1,497.86), fatigue (n = 139, ROR = 2.07), condition aggravated (n = 133, ROR = 4.94), asthenia (n = 122, ROR = 4.17), respiratory distress (n = 106, ROR = 2.35), muscular weakness (n = 104, ROR = 12.27), and unexpected therapeutic response (n = 98, ROR = 27.68) (Table 4). Among these, muscular weakness and unexpected therapeutic response aligned with the adverse reactions listed in the drug’s prescribing information. In addition to adverse effects like gait disturbances already noted in the prescribing information, this study also identified high-signal but rare AEs such as catheter site thrombosis, gastrostomy site leakage, and venous collapse (S3 Table). Although infrequent, these AEs exhibited strong signal intensities and merit further investigation. To further enhance clarity and reference value, we have provided a systematic summary table (S6 Table) categorizing the signals identified in this study into three groups: newly detected signals, signals consistent with the label, and signals not in the label but reported in existing literature.

**Table 4:** Top 30 adverse events by report number.

	SOC_English	PT_English	Case Reports	ROR (95% CI)	PRR (95% CI)	chisq	IC(IC025)
1	General disorders and administration site conditions	death	589	8.64 (7.93, 9.42)	7.74 (7.16, 8.37)	3504.11	2.95 (2.83)
2	General disorders and administration site conditions	disease progression	266	28.26 (24.97, 31.99)	26.8 (23.83, 30.14)	6591.96	4.74 (4.56)
3	General disorders and administration site conditions	drug ineffective	252	2.16 (1.9, 2.45)	2.1 (1.87, 2.36)	148.98	1.07 (0.89)
4	General disorders and administration site conditions	fatigue	139	2.07 (1.75, 2.45)	2.04 (1.74, 2.39)	74.34	1.03 (0.78)
5	General disorders and administration site conditions	condition aggravated	133	4.94 (4.15, 5.86)	4.83 (4.05, 5.76)	405.81	2.27 (2.02)
6	General disorders and administration site conditions	asthenia	122	4.17 (3.49, 5)	4.1 (3.44, 4.89)	286.96	2.03 (1.78)
7	General disorders and administration site conditions	therapeutic response unexpected	98	27.68 (22.66, 33.83)	27.16 (22.33, 33.04)	2460.23	4.76 (4.47)
8	General disorders and administration site conditions	gait disturbance	92	5.95 (4.84, 7.31)	5.85 (4.81, 7.12)	371.11	2.55 (2.25)
9	General disorders and administration site conditions	no adverse event	34	2.19 (1.56, 3.06)	2.18 (1.56, 3.04)	21.73	1.12 (0.64)
10	General disorders and administration site conditions	gait inability	23	5.72 (3.8, 8.61)	5.7 (3.78, 8.6)	89.03	2.51 (1.93)
11	General disorders and administration site conditions	adverse event	22	3.12 (2.05, 4.74)	3.11 (2.06, 4.69)	31.46	1.63 (1.04)
12	General disorders and administration site conditions	general physical health deterioration	20	2.11 (1.36, 3.27)	2.1 (1.36, 3.23)	11.6	1.07 (0.45)
13	General disorders and administration site conditions	energy increased	17	38.44 (23.85, 61.98)	38.32 (23.94, 61.34)	614.17	5.25 (4.58)
14	General disorders and administration site conditions	adverse drug reaction	17	2.09 (1.3, 3.37)	2.09 (1.31, 3.35)	9.66	1.06 (0.39)
15	General disorders and administration site conditions	catheter site swelling	5	69.18 (28.64, 167.07)	69.11 (28.61, 166.95)	332.02	6.1 (4.93)
16	General disorders and administration site conditions	catheter site pain	5	24.67 (10.25, 59.39)	24.64 (10.2, 59.52)	112.99	4.62 (3.46)
17	General disorders and administration site conditions	infusion site extravasation	5	8.04 (3.34, 19.32)	8.03 (3.32, 19.4)	30.73	3 (1.85)
18	General disorders and administration site conditions	catheter site thrombosis	5	165.74 (68.18, 402.93)	165.58 (68.54, 400)	797.16	7.33 (6.16)
19	General disorders and administration site conditions	infusion site pain	4	3.49 (1.31, 9.29)	3.48 (1.31, 9.27)	7.08	1.8 (0.53)
20	General disorders and administration site conditions	secretion discharge	4	3.6 (1.35, 9.59)	3.6 (1.35, 9.59)	7.49	1.85 (0.58)
21	General disorders and administration site conditions	loss of control of legs	3	13.1 (4.22, 40.68)	13.09 (4.2, 40.8)	33.44	3.71 (2.29)
22	Nervous system disorders	amyotrophic lateral sclerosis	182	1497.86 (1272.05, 1763.76)	1442.88 (1233.48, 1687.83)	213743.11	10.2 (9.97)
23	Nervous system disorders	aphasia	81	35.55 (28.53, 44.31)	34.99 (28.2, 43.41)	2661.09	5.12 (4.81)
24	Nervous system disorders	speech disorder	73	18.52 (14.69, 23.34)	18.26 (14.43, 23.1)	1188.52	4.19 (3.86)
25	Nervous system disorders	cerebral infarction	42	23.35 (17.23, 31.66)	23.16 (17.26, 31.08)	887.74	4.53 (4.09)
26	Nervous system disorders	balance disorder	21	3.08 (2.01, 4.73)	3.07 (1.99, 4.73)	29.42	1.62 (1.02)
27	Nervous system disorders	cerebral haemorrhage	15	5.53 (3.33, 9.19)	5.52 (3.32, 9.19)	55.48	2.46 (1.75)
28	Nervous system disorders	dysarthria	14	5.22 (3.09, 8.82)	5.21 (3.07, 8.84)	47.59	2.38 (1.65)
29	Nervous system disorders	muscle contractions involuntary	10	41.86 (22.46, 78)	41.78 (22.31, 78.23)	395.41	5.38 (4.52)
30	Nervous system disorders	dysstasia	9	3.85 (2, 7.41)	3.85 (2.02, 7.35)	18.96	1.94 (1.05)

The signal strength of AEs of Edaravone at the PTs level in FAERS database. ROR, reporting odds ratio; PRR, proportional reporting ratio; BCPNN, bayesian confidence propagation neural network; CI, confidence interval; 95%CI, 95% confidence interval; N, the number of reports;IC025, the lower limit of95% CI, for the IC.

### 3.3. Gender-based difference in significant signals with edaravone

To investigate whether gender affects the adverse reactions associated with edaravone, we separately analyzed the PTs of adverse events (AEs) between females and males. The results revealed gender differences at the PT level, as detailed in S1 and S2 Figs, S4 and S5 Tables. Compared to females, males reported a greater number of AEs (39 vs. 32). The data show that conditions such as drug ineffective (n = 119, ROR = 2.77), gait inability (n = 8, ROR = 4.88), catheter site pain (n = 3, ROR = 44.53), dependence on respirator (n = 3, ROR = 145.76), pneumonia aspiration (n = 13, ROR = 9.63), deep vein thrombosis (n = 7, ROR = 3.81), increased transaminases (n = 6, ROR = 6.7), and abnormal hepatic function (n = 8, ROR = 4.7) were more likely to occur in males. In contrast, females were more prone to experiencing infusion site pain (n = 3, ROR = 7.04), subarachnoid hemorrhage (n = 8, ROR = 4.7), aspiration (n = 3, ROR = 18.6), vasculitis (n = 4, ROR = 17.06), muscle twitching (n = 3, ROR = 6.41), and cardiac arrest (n = 8, ROR = 6.88).

These findings suggest significant gender differences in edaravone-related adverse events, which may be related to the potential protective role of sex hormones (especially estrogen and progesterone) in modulating ALS progression, potentially influencing neuroprotection and metabolic balance [24]. Studies have shown that estrogen can exert neuroprotective effects through antioxidant, anti-inflammatory, and neurotrophic mechanisms [25]. Experimental evidence from ALS transgenic mouse models further supports this notion: exogenous estrogen supplementation can prolong lifespan and delay motor dysfunction, whereas ovariectomy accelerates disease progression [26]. It is noteworthy that the primary mechanism of the free radical scavenger edaravone involves the reduction of oxidative stress, which may overlap or act synergistically with the antioxidant pathways of estrogen. The regulatory role of estrogen not only underscores its critical protective significance in the onset and progression of ALS but may also partly explain the influence of sex differences on the efficacy and adverse reactions of edaravone. Furthermore, male ALS patients exhibit more significant downregulation at the miRNA level compared to females, with gender-dependent differences [27]. The mechanisms underlying these gender differences remain unclear and warrant further investigation. These results may provide personalized recommendations for edaravone users, including gender-specific screening and treatment approaches.

## 4. Discussion

Our study revealed a higher incidence of edaravone-related AEs in males compared to females. This may be attributed to the primary indications of edaravone, such as acute ischemic stroke and ALS, which are more prevalent in males [28,29]. This observation is further supported by population epidemiological characteristics. Notably, severe adverse outcomes—including death, other serious events, disability, and life-threatening conditions—accounted for more than half of edaravone-related outcomes. A total of 78.84% of the reported cases involved serious outcomes, far exceeding half of all reports, highlighting significant safety concerns associated with edaravone use in real-world settings. Specifically (Table 1), the outcomes included death (n = 962, 55.48%), other serious adverse events (n = 375, 21.63%), disability (n = 11, 0.63%), and life-threatening conditions (n = 19, 1.10%). A case report described three patients with acute cerebral infarction who experienced cardiac arrest during edaravone treatment [30]. None of the patients had a prior history of cardiac disease, nor were they receiving other medications that could precipitate cardiac arrest. A representative case involved a 71-year-old patient who suffered cardiac arrest during treatment and subsequently died on the sixth day of hospitalization. Clinical observations suggest a possible association between the adverse event and edaravone use; however, a causal relationship remains to be confirmed. Therefore, close monitoring is warranted in clinical practice to promptly identify potential risks. Although significant adverse events such as death, disease progression, and drug ineffectiveness were observed in this study, it should be noted that these events may also result from the natural progression of ALS or cerebral infarction, rather than being entirely attributable to edaravone. Therefore, the causal relationship for these serious adverse events should be interpreted with caution. In clinical practice, safety data should be assessed in the context of patients’ disease characteristics and treatment background to allow a more accurate interpretation of drug safety. These data clearly indicate a high frequency of severe adverse outcomes related to edaravone, underscoring the need for vigilant monitoring of high-risk patients during treatment. This finding also calls for enhanced warnings and intervention strategies regarding serious adverse events to be incorporated into the drug’s labeling and risk management plans.

The annual distribution of edaravone-related AE reports showed a marked increase starting from Q2 2017, underscoring the importance of enhanced pharmacovigilance for this drug. Disproportionality analysis for PTs at the SOC level identified significant signals across 14 SOCs. Many of these adverse reactions, except for psychiatric disorders, were frequently reported during clinical trials and are mentioned in the drug’s labeling [31]. Several AEs cited in the prescribing information—such as liver dysfunction, disseminated intravascular coagulation syndrome, elevated blood urea, and acute lung injury—also exhibited significant signals in this study, further confirming the reliability of our findings. Among the major SOC signals, the most common were general disorders and administration site conditions, nervous system disorders, and respiratory, thoracic, and mediastinal disorders.

### 4.1. AEs related to general disorders and administration site conditions

The most frequently reported AEs in the category of general disorders and administration site conditions were death, disease progression, and drug inefficacy. Previous clinical studies have consistently identified risk signals for edaravone in this category. Deaths are often considered to result from disease progression or documented AEs [32]. ALS, a primary indication for edaravone, is an idiopathic neurodegenerative disease with approximately 50% of patients succumbing within 30 months of symptom onset [33]. While edaravone has shown efficacy in delaying disease progression in early-stage ALS patients, it may demonstrate diminished therapeutic effects in advanced cases, potentially contributing to drug inefficacy [34]. Additionally, several notable adverse signals were identified, such as unexpected therapeutic responses, catheter site swelling, catheter site pain, and catheter site thrombosis. These signals align with previous research and the drug’s labeling. Novel signals were also detected, including increased energy, secretory discharge, and loss of leg control. Although the unique nature of the disease may prevent precise reporting of some AEs during treatment, this does not constitute a valid reason to restrict edaravone use.

### 4.2. AEs related to nervous system disorders

Nervous system disorders represent a common category of edaravone-related AEs, complicating long-term treatment. ALS, one of edaravone’s key indications, exhibited prominent signals. Common nervous system-related AEs in our dataset included aphasia, speech disorders, cerebral infarction, balance disorders, cerebral hemorrhage, dysarthria, and involuntary muscle contractions. The onset and type of ALS significantly affect motor speech functions, with many patients already displaying marked dysarthria and speech disorders at diagnosis. Notably, ALS patients frequently regard the potential loss of speech as one of the most devastating symptoms [35]. Information on speech function deterioration is highly valued by patients, their families, and healthcare professionals [36]. Although studies have confirmed edaravone’s efficacy and safety in treating cerebral infarction and hemorrhage [37,38], our findings indicate a possibility of recurrent cerebral infarction or hemorrhage during edaravone treatment. The drug’s labeling also notes reports of cerebral embolism and intracerebral hemorrhage during or after infusion. While prior research has mentioned nervous system-related AEs [31], the drug’s labeling lacks detailed information on these events. Our data provide a comprehensive account of case numbers and signal values for edaravone-related nervous system AEs, offering insights for identifying and addressing these side effects during clinical practice.

### 4.3. AEs related to respiratory, thoracic, and mediastinal disorders

In this category, some PTs not listed in the drug labeling were identified, including ventilator dependence, aspiration, respiratory arrest, abnormal breathing, and sputum retention. Respiratory issues linked to respiratory muscle weakness are major causes of morbidity and mortality in ALS patients [39,40]. Therefore, alongside edaravone treatment, non-pharmacological respiratory therapies, should be employed to stabilize respiratory function in ALS patients [40]. The drug labeling also highlights the possibility of acute lung injury (incidence unknown) during edaravone use, with symptoms such as fever, cough, dyspnea, and abnormal chest X-ray findings. Our real-world data analysis also detected positive signals for acute lung injury, including respiratory failure and respiratory distress. In clinical practice, it is crucial to monitor for respiratory, thoracic, and mediastinal AEs during edaravone treatment, especially acute lung injury. Therapy should be promptly discontinued, and symptomatic management initiated if such symptoms occur.

### 4.4. AEs in other system organ classes

Our safety analysis revealed that edaravone-related AEs might also affect other organ systems. Infections and infestations associated with edaravone use included aspiration pneumonia, device-related infections, and injection site infections, with numerous case reports and positive signals. The drug labeling advises caution in patients with infections to prevent systemic deterioration leading to acute kidney injury or worsening renal dysfunction. In laboratory findings, elevated transaminases, blood urea, and cystatin C levels were observed. Hepatobiliary disorders included liver dysfunction, while hematologic and lymphatic disorders included disseminated intravascular coagulation (DIC). These findings align with the drug’s labeling. In cases of severe AEs, such as acute renal failure, nephrotic syndrome, liver dysfunction, or DIC, close monitoring and thorough observation are necessary. Therapy should be discontinued if abnormalities are detected, and appropriate measures should be taken. Additionally, several AEs not listed in the labeling were identified, including dysphagia, gastric fistula, and gastric ulcer bleeding, with notable case reports and positive signals. These findings underscore the importance of closely monitoring AEs during edaravone treatment.

### 4.5. Clinical practice and future research on the detection of novel adverse signals

The identification of novel adverse Signals provides supplementary evidence for rational prescribing, enabling clinicians to more thoroughly balance the therapeutic benefits and potential risks of edaravone. For newly identified adverse signals, such as catheter site thrombosis, clinicians should carefully assess their potential clinical impact and implement appropriate monitoring measures. For example, patients receiving intravenous edaravone should undergo regular evaluation of the catheter site for swelling, pain, or abnormal venous changes. Patients with potential hypercoagulable or bleeding risks should have periodic coagulation function assessments. Additionally, clinicians should monitor treatment response, energy levels, changes in secretions, and lower limb motor function to guide dose adjustments or individualized therapy. This contributes to improving the appropriateness and safety of its clinical use. In addition, these findings support the development of targeted monitoring protocols and risk management strategies for high-risk populations, guiding clinicians to enhance the monitoring of relevant clinical indicators and implement timely interventions. Moreover, emerging signals help to establish standardized AE management pathways. By integrating emergency procedures with clinical systems, the efficiency of response and handling can be improved, ultimately reducing the risk of drug-related harm.

Future studies should focus on dedicated pharmacovigilance investigations to clarify the causality and occurrence of observed adverse reactions. In-depth exploration of the mechanisms underlying edaravone-related side effects is also needed. Laboratory-based studies may help elucidate the potential molecular pathways involved in these ADRs and deepen understanding of the drug’s pharmacological mechanisms. At the same time, efforts should be made to identify biomarkers and clinical predictors, and to develop risk prediction models for identifying high-risk individuals. When sufficient evidence is available, signal evaluation reports should be submitted to regulatory agencies to inform updates to product labeling or safety warnings, thereby enhancing medication safety.

### 4.6. Limitations

Despite providing robust evidence for evaluating the safety of edaravone, our study has some limitations. The inherent biases of spontaneous reporting systems may affect the observed incidence and severity of AEs. First, the FAERS database relies on voluntary reporting, which often results in incomplete data submission and may lead to either overestimation or underestimation of AE incidence. Additionally, both patients and healthcare professionals tend to report more severe AEs, while mild or commonly occurring events are more likely to be overlooked. This reporting bias may artificially inflate the proportion of severe AEs, potentially leading to an overestimation of their overall severity. A clinical study including 194 ALS patients reported that 16% of participants experienced potential adverse reactions, primarily infusion-site infections and allergic responses [41]. In contrast, analysis of FAERS-based data showed a markedly higher proportion of serious events, with a total of 375 other serious adverse events reported, accounting for 21.63% of cases. Lastly, while our findings highlight safety signals, they do not establish a causal relationship between the drug and AEs. In clinical practice, edaravone treatment is frequently accompanied by comorbidities and polypharmacy, making it difficult to determine its direct pathogenic role. Moreover, real-world data are characterized by numerous and complex confounding factors, which further complicate the accurate isolation of edaravone’s independent causal effects, thereby posing significant challenges to causal inference.

Nevertheless, pharmacovigilance analyses using the FAERS database enable early detection of drug safety signals in diverse populations. This approach greatly improves post-marketing surveillance and facilitates timely interventions to protect public health. Future efforts to establish causality could include the following approaches:At the methodological level, innovation in causal inference techniques is needed to effectively control for confounding factors and precisely evaluate causal effects. At the biological mechanism level, multi-omics validation can be integrated to provide biological support for causal associations. At the research design level, optimization of study designs should be pursued to systematically analyze the causal relationship between edaravone use and the occurrence of AEs.

## 5. Conclusions

In summary, this study analyzed safety data on edaravone from the FAERS database, confirming the potential risks associated with edaravone use. Additionally, previously unreported events and significant signal intensities were identified, with general disorders and administration site conditions being the most common categories. Future clinical trials and epidemiological studies are needed to comprehensively and accurately assess the safety risks of edaravone. Overall, the findings provide valuable insights for clinical drug monitoring and safety evaluation of edaravone, offering essential references for regulatory authorities and healthcare institutions in formulating management strategies.

## Supporting information

S1 Fig PTs distribution diagram under four common SOC levels (Female). (TIF)

S2 Fig PTs distribution diagram under four common SOC levels (Male). (TIF)

S1 Table Two-by-two contingency table for analyses. (DOC)

S2 Table Summary of major algorithms used for signal detection. (DOCX)

S3 Table The signal strength of AEs of Edaravone at the PTs level in FAERS database. (DOC)

S4 Table The signal strength of AEs of Edaravone at the PTs level in FAERS database(Female). (DOC)

S5 Table The signal strength of AEs of Edaravone at the PTs level in FAERS database(Male). (DOC)

S6 Table Classification and Statistical Analysis of AEs of Edaravone. (DOC)
