# Supplementary figures and images for: Safety assessment of Edaravone: A real-world adverse event analysis based on the FAERS Database
Source: PLoS One. 2025 Oct 23;20(10):e0335362. doi: 10.1371/journal.pone.0335362 (PMC12548856; doi:10.1371/journal.pone.0335362)

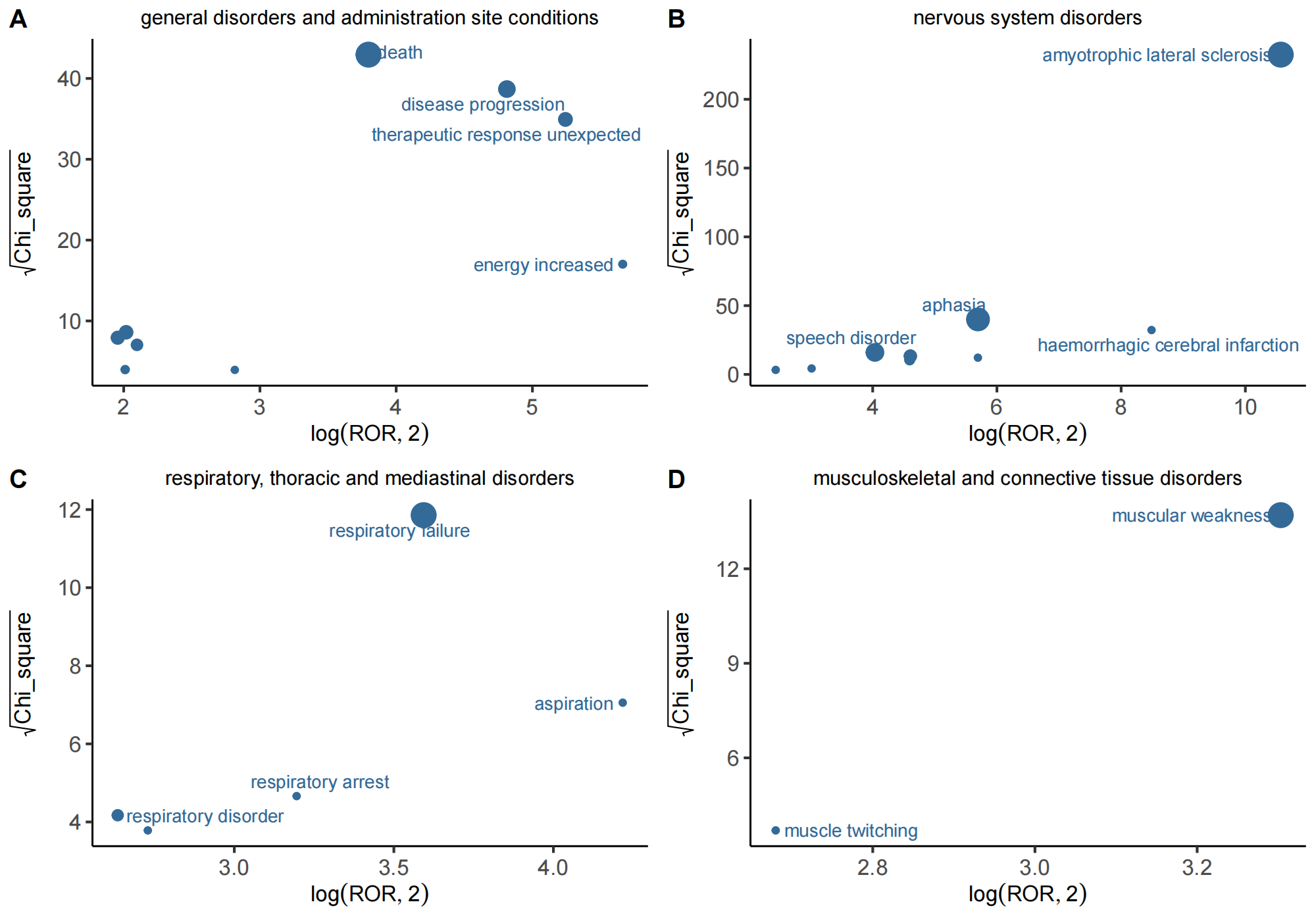

Supplement: S1 Fig — (TIF) [file pone.0335362.s001.tif]

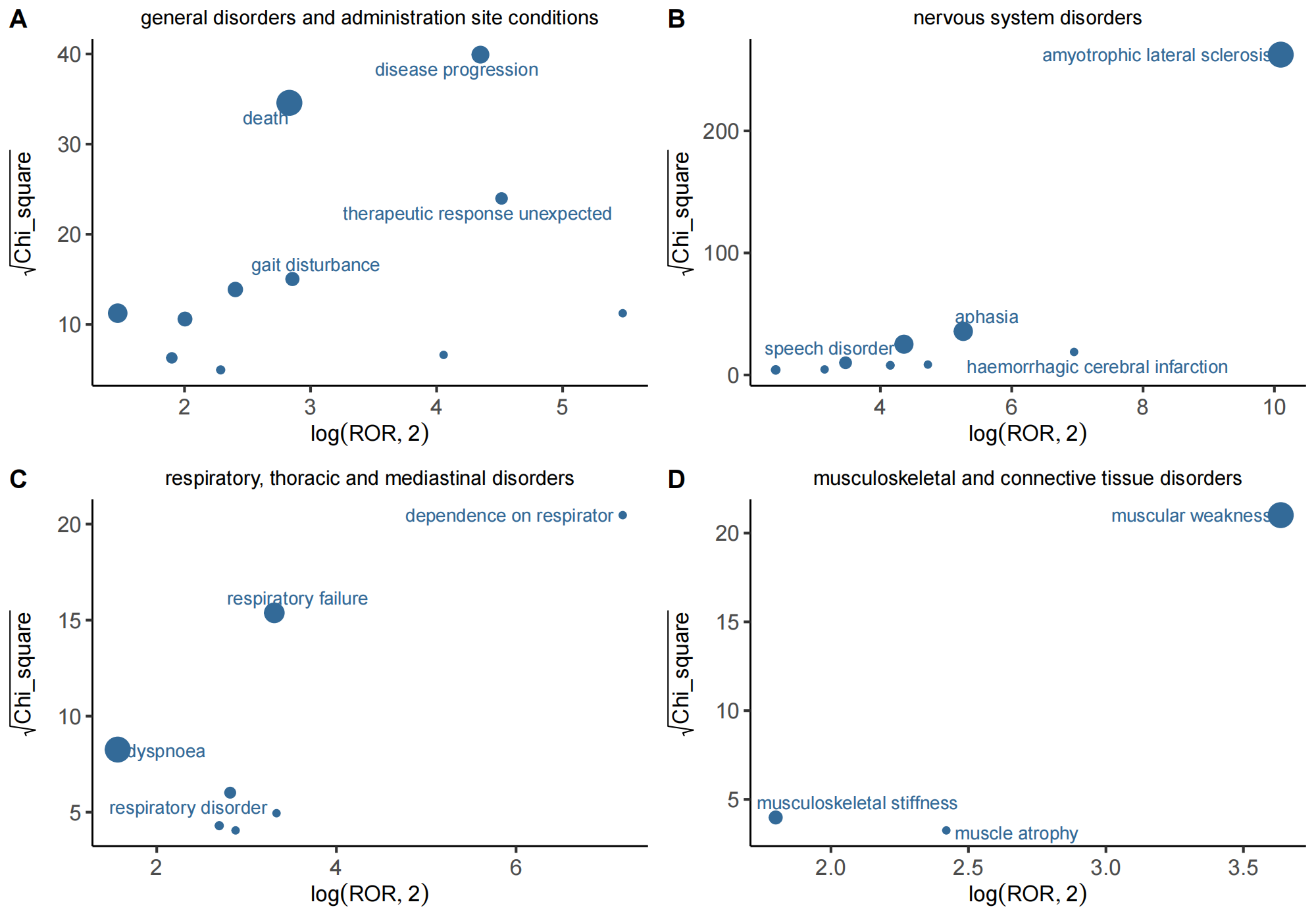

Supplement: S2 Fig — (TIF) [file pone.0335362.s002.tif]
