# Supplementary material for: Safety assessment of Edaravone: A real-world adverse event analysis based on the FAERS Database
Source: PLoS One. 2025 Oct 23;20(10):e0335362. doi: 10.1371/journal.pone.0335362 (PMC12548856; doi:10.1371/journal.pone.0335362)
Supplement: S1 Table — (DOC) [file pone.0335362.s003.doc]

**S1 Table. Two-by-two contingency table for analyses.**

| Drug | Target adverse events | Non-target adverse events | total |
| --- | --- | --- | --- |
| edaravone | a | b | a+b |
| Non-edaravone | c | d | c+d |
| Total | a+c | b+d | a+b+c+d |
