# Supplementary material for: Safety assessment of Edaravone: A real-world adverse event analysis based on the FAERS Database
Source: PLoS One. 2025 Oct 23;20(10):e0335362. doi: 10.1371/journal.pone.0335362 (PMC12548856; doi:10.1371/journal.pone.0335362)
Supplement: S2 Table — (DOCX) [file pone.0335362.s004.docx]

S2 Table. Summary of major algorithms used for signal detection.

| Method | Formula | Threshold |
| --- | --- | --- |
| ROR | $ROR=\frac{a}{b}\div\frac{c}{d}$ = $\frac{\mathrm{ad}}{\mathrm{bc}}$  ${95\%CI=e}^{lnROR\pm1.96\sqrt{\frac{1}{a} +\frac{1}{b} + \frac{1}{c} + \frac{1}{d}}}$ | a ≥ 3 and 95% CI (lower limit) > 1 |
| PRR | $PRR=\frac{a}{a+c}\div\frac{b}{b+d}$ = $\frac{a(b+d)}{(a+c)b}$  ${95\%CI=e}^{lnROR\pm1.96\sqrt{\frac{1}{a} +\frac{1}{b} + \frac{1}{c} + \frac{1}{d}}}$ | a ≥ 3 and 95% CI (lower limit) > 1 |
| BCPNN | IC = log2a (a+b + c + d)/((a+c) (a+b))  IC025 = eln(IC)−1.96(1/a+1/b+1/c+1/d)^0.5 | IC025 > 0, a ≥3 |

Equation:ROR, PRR, and BCPNN methods, formulas, and thresholds. ROR, reporting odds ratio; PRR, proportional reporting ratio; BCPNN, bayesian confidence propagation neural network; CI, confidence interval; 95%CI, 95% confidence interval; N, the number of reports;IC025, the lower limit of95% CI, for the IC.
