# Supplementary material for: Safety assessment of Edaravone: A real-world adverse event analysis based on the FAERS Database
Source: PLoS One. 2025 Oct 23;20(10):e0335362. doi: 10.1371/journal.pone.0335362 (PMC12548856; doi:10.1371/journal.pone.0335362)
Supplement: S3 Table — (DOC) [file pone.0335362.s005.doc]

**S3 Table.The signal strength of AEs of Edaravone at the PTs level in FAERS database.**

|  | **soc_english** | **pt_english** | **Case Reports** | **ROR**  **(95% CI)** | **PRR**  **(95% CI)** | **chisq** | **IC(IC025)** |
| --- | --- | --- | --- | --- | --- | --- | --- |
| 1 | general disorders and administration site conditions | death | 589 | 8.64(7.93, 9.42) | 7.74(7.16, 8.37) | 3504.11 | 2.95(2.83) |
| 2 | general disorders and administration site conditions | disease progression | 266 | 28.26(24.97, 31.99) | 26.8(23.83, 30.14) | 6591.96 | 4.74(4.56) |
| 3 | general disorders and administration site conditions | drug ineffective | 252 | 2.16(1.9, 2.45) | 2.1(1.87, 2.36) | 148.98 | 1.07(0.89) |
| 4 | general disorders and administration site conditions | fatigue | 139 | 2.07(1.75, 2.45) | 2.04(1.74, 2.39) | 74.34 | 1.03(0.78) |
| 5 | general disorders and administration site conditions | condition aggravated | 133 | 4.94(4.15, 5.86) | 4.83(4.05, 5.76) | 405.81 | 2.27(2.02) |
| 6 | general disorders and administration site conditions | asthenia | 122 | 4.17(3.49, 5) | 4.1(3.44, 4.89) | 286.96 | 2.03(1.78) |
| 7 | general disorders and administration site conditions | therapeutic response unexpected | 98 | 27.68(22.66, 33.83) | 27.16(22.33, 33.04) | 2460.23 | 4.76(4.47) |
| 8 | general disorders and administration site conditions | gait disturbance | 92 | 5.95(4.84, 7.31) | 5.85(4.81, 7.12) | 371.11 | 2.55(2.25) |
| 9 | general disorders and administration site conditions | no adverse event | 34 | 2.19(1.56, 3.06) | 2.18(1.56, 3.04) | 21.73 | 1.12(0.64) |
| 10 | general disorders and administration site conditions | gait inability | 23 | 5.72(3.8, 8.61) | 5.7(3.78, 8.6) | 89.03 | 2.51(1.93) |
| 11 | general disorders and administration site conditions | adverse event | 22 | 3.12(2.05, 4.74) | 3.11(2.06, 4.69) | 31.46 | 1.63(1.04) |
| 12 | general disorders and administration site conditions | general physical health deterioration | 20 | 2.11(1.36, 3.27) | 2.1(1.36, 3.23) | 11.6 | 1.07(0.45) |
| 13 | general disorders and administration site conditions | energy increased | 17 | 38.44(23.85, 61.98) | 38.32(23.94, 61.34) | 614.17 | 5.25(4.58) |
| 14 | general disorders and administration site conditions | adverse drug reaction | 17 | 2.09(1.3, 3.37) | 2.09(1.31, 3.35) | 9.66 | 1.06(0.39) |
| 15 | general disorders and administration site conditions | catheter site swelling | 5 | 69.18(28.64, 167.07) | 69.11(28.61, 166.95) | 332.02 | 6.1(4.93) |
| 16 | general disorders and administration site conditions | catheter site pain | 5 | 24.67(10.25, 59.39) | 24.64(10.2, 59.52) | 112.99 | 4.62(3.46) |
| 17 | general disorders and administration site conditions | infusion site extravasation | 5 | 8.04(3.34, 19.32) | 8.03(3.32, 19.4) | 30.73 | 3(1.85) |
| 18 | general disorders and administration site conditions | catheter site thrombosis | 5 | 165.74(68.18, 402.93) | 165.58(68.54, 400) | 797.16 | 7.33(6.16) |
| 19 | general disorders and administration site conditions | infusion site pain | 4 | 3.49(1.31, 9.29) | 3.48(1.31, 9.27) | 7.08 | 1.8(0.53) |
| 20 | general disorders and administration site conditions | secretion discharge | 4 | 3.6(1.35, 9.59) | 3.6(1.35, 9.59) | 7.49 | 1.85(0.58) |
| 21 | general disorders and administration site conditions | loss of control of legs | 3 | 13.1(4.22, 40.68) | 13.09(4.2, 40.8) | 33.44 | 3.71(2.29) |
| 22 | nervous system disorders | amyotrophic lateral sclerosis | 182 | 1497.86(1272.05, 1763.76) | 1442.88(1233.48, 1687.83) | 213743.11 | 10.2(9.97) |
| 23 | nervous system disorders | aphasia | 81 | 35.55(28.53, 44.31) | 34.99(28.2, 43.41) | 2661.09 | 5.12(4.81) |
| 24 | nervous system disorders | speech disorder | 73 | 18.52(14.69, 23.34) | 18.26(14.43, 23.1) | 1188.52 | 4.19(3.86) |
| 25 | nervous system disorders | cerebral infarction | 42 | 23.35(17.23, 31.66) | 23.16(17.26, 31.08) | 887.74 | 4.53(4.09) |
| 26 | nervous system disorders | balance disorder | 21 | 3.08(2.01, 4.73) | 3.07(1.99, 4.73) | 29.42 | 1.62(1.02) |
| 27 | nervous system disorders | cerebral haemorrhage | 15 | 5.53(3.33, 9.19) | 5.52(3.32, 9.19) | 55.48 | 2.46(1.75) |
| 28 | nervous system disorders | dysarthria | 14 | 5.22(3.09, 8.82) | 5.21(3.07, 8.84) | 47.59 | 2.38(1.65) |
| 29 | nervous system disorders | muscle contractions involuntary | 10 | 41.86(22.46, 78) | 41.78(22.31, 78.23) | 395.41 | 5.38(4.52) |
| 30 | nervous system disorders | dysstasia | 9 | 3.85(2, 7.41) | 3.85(2.02, 7.35) | 18.96 | 1.94(1.05) |
| 31 | nervous system disorders | dysgraphia | 9 | 16.72(8.69, 32.18) | 16.69(8.74, 31.87) | 132.44 | 4.06(3.16) |
| 32 | nervous system disorders | subarachnoid haemorrhage | 7 | 8.19(3.9, 17.2) | 8.18(3.88, 17.23) | 44.08 | 3.03(2.03) |
| 33 | nervous system disorders | hypokinesia | 6 | 4.69(2.1, 10.44) | 4.68(2.1, 10.45) | 17.36 | 2.23(1.16) |
| 34 | nervous system disorders | paralysis | 6 | 5.52(2.48, 12.3) | 5.51(2.47, 12.31) | 22.16 | 2.46(1.39) |
| 35 | nervous system disorders | haemorrhagic cerebral infarction | 4 | 89.31(33.28, 239.69) | 89.24(33.49, 237.78) | 344.17 | 6.46(5.18) |
| 36 | nervous system disorders | fine motor skill dysfunction | 4 | 12.52(4.69, 33.4) | 12.51(4.7, 33.33) | 42.28 | 3.64(2.38) |
| 37 | nervous system disorders | motor dysfunction | 4 | 4.89(1.83, 13.03) | 4.88(1.83, 13) | 12.34 | 2.29(1.02) |
| 38 | nervous system disorders | myasthenia gravis | 3 | 4.33(1.39, 13.42) | 4.32(1.39, 13.46) | 7.66 | 2.11(0.7) |
| 39 | respiratory, thoracic and mediastinal disorders | dyspnoea | 106 | 2.35(1.94, 2.85) | 2.32(1.91, 2.82) | 80.2 | 1.21(0.94) |
| 40 | respiratory, thoracic and mediastinal disorders | respiratory failure | 56 | 10.39(7.98, 13.53) | 10.29(7.98, 13.28) | 469.28 | 3.36(2.98) |
| 41 | respiratory, thoracic and mediastinal disorders | respiratory disorder | 28 | 11.93(8.23, 17.3) | 11.87(8.18, 17.23) | 278.32 | 3.57(3.04) |
| 42 | respiratory, thoracic and mediastinal disorders | dysphonia | 10 | 2.05(1.1, 3.82) | 2.05(1.09, 3.84) | 5.38 | 1.04(0.18) |
| 43 | respiratory, thoracic and mediastinal disorders | choking | 8 | 5.05(2.52, 10.1) | 5.04(2.54, 10.01) | 25.89 | 2.33(1.39) |
| 44 | respiratory, thoracic and mediastinal disorders | respiratory distress | 7 | 3.45(1.65, 7.25) | 3.45(1.64, 7.27) | 12.19 | 1.79(0.79) |
| 45 | respiratory, thoracic and mediastinal disorders | aphonia | 7 | 5.92(2.82, 12.43) | 5.91(2.81, 12.45) | 28.56 | 2.56(1.56) |
| 46 | respiratory, thoracic and mediastinal disorders | dependence on respirator | 7 | 163.26(77.06, 345.89) | 163.03(77.41, 343.34) | 1099.08 | 7.31(6.3) |
| 47 | respiratory, thoracic and mediastinal disorders | aspiration | 6 | 7.78(3.49, 17.34) | 7.78(3.48, 17.38) | 35.39 | 2.96(1.89) |
| 48 | respiratory, thoracic and mediastinal disorders | acute respiratory failure | 6 | 3.65(1.64, 8.13) | 3.65(1.63, 8.15) | 11.52 | 1.87(0.8) |
| 49 | respiratory, thoracic and mediastinal disorders | respiratory arrest | 6 | 3.77(1.69, 8.39) | 3.76(1.68, 8.4) | 12.17 | 1.91(0.84) |
| 50 | respiratory, thoracic and mediastinal disorders | respiration abnormal | 4 | 5.69(2.13, 15.18) | 5.69(2.14, 15.16) | 15.45 | 2.51(1.24) |
| 51 | respiratory, thoracic and mediastinal disorders | asphyxia | 3 | 3.9(1.26, 12.11) | 3.9(1.25, 12.16) | 6.47 | 1.96(0.55) |
| 52 | respiratory, thoracic and mediastinal disorders | sputum retention | 3 | 39.43(12.67, 122.74) | 39.41(12.64, 122.83) | 111.62 | 5.29(3.87) |
| 53 | infections and infestations | pneumonia aspiration | 26 | 12.85(8.74, 18.9) | 12.79(8.64, 18.93) | 282.1 | 3.67(3.13) |
| 54 | infections and infestations | sepsis | 18 | 2.03(1.28, 3.22) | 2.02(1.26, 3.23) | 9.33 | 1.02(0.37) |
| 55 | infections and infestations | device related infection | 17 | 14.43(8.96, 23.24) | 14.38(8.98, 23.02) | 211.27 | 3.84(3.17) |
| 56 | infections and infestations | injection site infection | 7 | 29.99(14.27, 63.06) | 29.95(14.22, 63.08) | 194.98 | 4.9(3.89) |
| 57 | infections and infestations | catheter site infection | 7 | 29.69(14.12, 62.42) | 29.65(14.08, 62.44) | 192.91 | 4.88(3.88) |
| 58 | infections and infestations | device related sepsis | 3 | 16.05(5.17, 49.86) | 16.04(5.15, 49.99) | 42.21 | 4(2.58) |
| 59 | vascular disorders | poor venous access | 14 | 14.17(8.38, 23.95) | 14.13(8.32, 23.99) | 170.46 | 3.82(3.09) |
| 60 | vascular disorders | deep vein thrombosis | 13 | 3.18(1.85, 5.48) | 3.17(1.83, 5.49) | 19.37 | 1.67(0.91) |
| 61 | vascular disorders | haemorrhagic infarction | 4 | 92.22(34.35, 247.56) | 92.15(34.58, 245.53) | 355.49 | 6.51(5.23) |
| 62 | vascular disorders | vasculitis | 4 | 4.49(1.68, 11.97) | 4.49(1.69, 11.96) | 10.83 | 2.16(0.9) |
| 63 | vascular disorders | vein collapse | 4 | 48.12(17.98, 128.73) | 48.08(18.04, 128.11) | 183.01 | 5.58(4.31) |
| 64 | musculoskeletal and connective tissue disorders | muscular weakness | 104 | 12.27(10.11, 14.91) | 12.04(9.9, 14.65) | 1052.45 | 3.59(3.31) |
| 65 | musculoskeletal and connective tissue disorders | mobility decreased | 30 | 4.98(3.48, 7.13) | 4.96(3.49, 7.06) | 94.77 | 2.31(1.8) |
| 66 | musculoskeletal and connective tissue disorders | muscle twitching | 8 | 4.74(2.37, 9.49) | 4.74(2.39, 9.41) | 23.58 | 2.24(1.3) |
| 67 | musculoskeletal and connective tissue disorders | muscle atrophy | 8 | 8.93(4.46, 17.87) | 8.91(4.49, 17.69) | 56.13 | 3.15(2.21) |
| 68 | musculoskeletal and connective tissue disorders | limb discomfort | 7 | 2.39(1.14, 5.02) | 2.39(1.13, 5.03) | 5.67 | 1.26(0.26) |
| 69 | investigations | transaminases increased | 6 | 3.2(1.44, 7.13) | 3.2(1.43, 7.15) | 9.07 | 1.68(0.61) |
| 70 | investigations | pulmonary function test decreased | 4 | 9.62(3.6, 25.65) | 9.61(3.61, 25.61) | 30.8 | 3.26(2) |
| 71 | investigations | forced vital capacity decreased | 4 | 54.27(20.28, 145.26) | 54.23(20.35, 144.49) | 207.22 | 5.75(4.48) |
| 72 | investigations | blood urea increased | 3 | 3.13(1.01, 9.7) | 3.12(1, 9.72) | 4.33 | 1.64(0.23) |
| 73 | investigations | cystatin c increased | 3 | 329.07(103.07, 1050.59) | 328.87(103.47, 1045.3) | 932.4 | 8.29(6.83) |
| 74 | injury, poisoning and procedural complications | fall | 74 | 2.77(2.2, 3.48) | 2.74(2.17, 3.47) | 82.25 | 1.45(1.13) |
| 75 | injury, poisoning and procedural complications | incorrect product administration duration | 9 | 3.42(1.78, 6.58) | 3.42(1.79, 6.53) | 15.38 | 1.77(0.88) |
| 76 | injury, poisoning and procedural complications | fracture | 7 | 3.91(1.86, 8.22) | 3.91(1.86, 8.23) | 15.16 | 1.97(0.97) |
| 77 | injury, poisoning and procedural complications | discontinued product administered | 4 | 109.24(40.64, 293.63) | 109.15(40.97, 290.83) | 421.42 | 6.75(5.47) |
| 78 | gastrointestinal disorders | dysphagia | 35 | 4.77(3.42, 6.66) | 4.75(3.4, 6.63) | 103.61 | 2.25(1.77) |
| 79 | gastrointestinal disorders | gastric fistula | 5 | 124.31(51.28, 301.34) | 124.18(51.4, 299.98) | 599.26 | 6.93(5.76) |
| 80 | gastrointestinal disorders | salivary hypersecretion | 4 | 5.14(1.93, 13.72) | 5.14(1.93, 13.7) | 13.33 | 2.36(1.1) |
| 81 | gastrointestinal disorders | gastric ulcer haemorrhage | 3 | 7.62(2.45, 23.65) | 7.61(2.44, 23.72) | 17.22 | 2.93(1.51) |
| 82 | psychiatric disorders | eating disorder | 6 | 3.25(1.46, 7.23) | 3.24(1.45, 7.24) | 9.32 | 1.7(0.63) |
| 83 | metabolism and nutrition disorders | feeding disorder | 5 | 2.7(1.12, 6.49) | 2.7(1.12, 6.52) | 5.34 | 1.43(0.28) |
| 84 | hepatobiliary disorders | hepatic function abnormal | 16 | 5.45(3.33, 8.9) | 5.43(3.33, 8.86) | 57.88 | 2.44(1.75) |
| 85 | cardiac disorders | cardiac arrest | 16 | 2.82(1.73, 4.62) | 2.82(1.73, 4.6) | 18.79 | 1.49(0.81) |
| 86 | blood and lymphatic system disorders | disseminated intravascular coagulation | 4 | 4.49(1.68, 11.96) | 4.48(1.68, 11.94) | 10.82 | 2.16(0.9) |

ROR, reporting odds ratio; PRR, proportional reporting ratio; BCPNN, bayesian confidence propagation neural network; CI, confidence interval; 95%CI, 95% confidence interval; N, the number of reports;IC025, the lower limit of95% CI, for the IC.
