# Supplementary material for: Safety assessment of Edaravone: A real-world adverse event analysis based on the FAERS Database
Source: PLoS One. 2025 Oct 23;20(10):e0335362. doi: 10.1371/journal.pone.0335362 (PMC12548856; doi:10.1371/journal.pone.0335362)
Supplement: S4 Table — (DOC) [file pone.0335362.s006.doc]

S4 Table.The signal strength of AEs of Edaravone at the PTs level in FAERS database(Female).

|  | **soc_english** | **pt_english** | **Case Reports** | **ROR(95% CI)** | **PRR(95% CI)** | **chisq** | **IC(IC025)** |
| --- | --- | --- | --- | --- | --- | --- | --- |
| 1 | general disorders and administration site conditions | death | 177 | 13.9(11.87, 16.27) | 12.24(10.67, 14.04) | 1844.37 | 3.61(3.39) |
| 2 | general disorders and administration site conditions | disease progression | 60 | 28.1(21.69, 36.41) | 26.92(20.86, 34.73) | 1496.69 | 4.75(4.38) |
| 3 | general disorders and administration site conditions | therapeutic response unexpected | 35 | 37.85(27.05, 52.97) | 36.91(26.45, 51.51) | 1220.19 | 5.2(4.72) |
| 4 | general disorders and administration site conditions | asthenia | 33 | 4.05(2.87, 5.72) | 3.97(2.85, 5.54) | 73.9 | 1.99(1.5) |
| 5 | general disorders and administration site conditions | condition aggravated | 30 | 3.88(2.7, 5.57) | 3.82(2.68, 5.44) | 62.78 | 1.93(1.42) |
| 6 | general disorders and administration site conditions | gait disturbance | 20 | 4.28(2.75, 6.66) | 4.23(2.75, 6.51) | 49.55 | 2.08(1.46) |
| 7 | general disorders and administration site conditions | adverse event | 7 | 4.03(1.92, 8.46) | 4.01(1.9, 8.45) | 15.83 | 2(1) |
| 8 | general disorders and administration site conditions | energy increased | 6 | 50.66(22.68, 113.14) | 50.44(22.58, 112.66) | 289.64 | 5.65(4.58) |
| 9 | general disorders and administration site conditions | infusion site pain | 3 | 7.04(2.27, 21.88) | 7.03(2.26, 21.91) | 15.52 | 2.81(1.4) |
| 10 | nervous system disorders | amyotrophic lateral sclerosis | 41 | 1519.75(1094.78, 2109.69) | 1474.57(1077.63, 2017.72) | 54029.31 | 10.37(9.9) |
| 11 | nervous system disorders | aphasia | 33 | 51.82(36.66, 73.24) | 50.6(36.26, 70.61) | 1598.76 | 5.66(5.16) |
| 12 | nervous system disorders | speech disorder | 18 | 16.4(10.3, 26.11) | 16.19(10.31, 25.41) | 256.49 | 4.02(3.36) |
| 13 | nervous system disorders | cerebral infarction | 8 | 24.36(12.15, 48.84) | 24.23(12.2, 48.11) | 177.83 | 4.6(3.65) |
| 14 | nervous system disorders | subarachnoid haemorrhage | 5 | 24.17(10.03, 58.2) | 24.08(9.97, 58.17) | 110.42 | 4.59(3.43) |
| 15 | nervous system disorders | cerebral haemorrhage | 3 | 5.42(1.74, 16.83) | 5.41(1.74, 16.86) | 10.78 | 2.43(1.02) |
| 16 | nervous system disorders | haemorrhagic cerebral infarction | 3 | 359.4(113.93, 1133.79) | 358.62(115.06, 1117.74) | 1040.16 | 8.45(7) |
| 17 | nervous system disorders | muscle contractions involuntary | 3 | 51.76(16.64, 161.07) | 51.65(16.57, 160.98) | 148.41 | 5.68(4.27) |
| 18 | nervous system disorders | hypokinesia | 3 | 8.09(2.6, 25.12) | 8.07(2.59, 25.15) | 18.58 | 3.01(1.6) |
| 19 | respiratory, thoracic and mediastinal disorders | respiratory failure | 14 | 12.07(7.13, 20.44) | 11.96(7.05, 20.3) | 140.59 | 3.58(2.84) |
| 20 | respiratory, thoracic and mediastinal disorders | respiratory disorder | 4 | 6.21(2.32, 16.56) | 6.19(2.32, 16.49) | 17.41 | 2.63(1.36) |
| 21 | respiratory, thoracic and mediastinal disorders | aspiration | 3 | 18.6(5.99, 57.78) | 18.56(5.95, 57.85) | 49.77 | 4.21(2.79) |
| 22 | respiratory, thoracic and mediastinal disorders | respiratory arrest | 3 | 9.16(2.95, 28.45) | 9.14(2.93, 28.49) | 21.74 | 3.19(1.77) |
| 23 | respiratory, thoracic and mediastinal disorders | choking | 3 | 6.63(2.14, 20.6) | 6.62(2.12, 20.63) | 14.32 | 2.73(1.31) |
| 24 | infections and infestations | device related infection | 4 | 13.95(5.23, 37.25) | 13.92(5.22, 37.09) | 47.91 | 3.8(2.53) |
| 25 | infections and infestations | injection site infection | 3 | 45.36(14.58, 141.09) | 45.26(14.52, 141.07) | 129.38 | 5.5(4.08) |
| 26 | infections and infestations | catheter site infection | 3 | 44.76(14.39, 139.25) | 44.67(14.33, 139.23) | 127.62 | 5.48(4.06) |
| 27 | vascular disorders | vasculitis | 4 | 17.06(6.39, 45.55) | 17.01(6.38, 45.32) | 60.22 | 4.09(2.82) |
| 28 | vascular disorders | poor venous access | 4 | 14.96(5.6, 39.93) | 14.92(5.6, 39.75) | 51.88 | 3.9(2.63) |
| 29 | musculoskeletal and connective tissue disorders | muscular weakness | 24 | 9.87(6.59, 14.77) | 9.71(6.56, 14.37) | 187.75 | 3.28(2.71) |
| 30 | musculoskeletal and connective tissue disorders | muscle twitching | 3 | 6.41(2.07, 19.92) | 6.4(2.05, 19.95) | 13.67 | 2.68(1.26) |
| 31 | gastrointestinal disorders | dysphagia | 15 | 7.99(4.8, 13.3) | 7.92(4.76, 13.18) | 90.72 | 2.98(2.27) |
| 32 | cardiac disorders | cardiac arrest | 8 | 6.88(3.43, 13.79) | 6.85(3.45, 13.6) | 39.95 | 2.77(1.83) |

ROR, reporting odds ratio; PRR, proportional reporting ratio; BCPNN, bayesian confidence propagation neural network; CI, confidence interval; 95%CI, 95% confidence interval; N, the number of reports;IC025, the lower limit of95% CI, for the IC.
