# Supplementary material for: Safety assessment of Edaravone: A real-world adverse event analysis based on the FAERS Database
Source: PLoS One. 2025 Oct 23;20(10):e0335362. doi: 10.1371/journal.pone.0335362 (PMC12548856; doi:10.1371/journal.pone.0335362)
Supplement: S5 Table — (DOC) [file pone.0335362.s007.doc]

**S5 Table.The signal strength of AEs of Edaravone at the PTs level in FAERS database(Male).**

|  | **soc_english** | **pt_english** | **Case Reports** | **ROR**  **(95% CI)** | **PRR**  **(95% CI)** | **chisq** | **IC(IC025)** |
| --- | --- | --- | --- | --- | --- | --- | --- |
| 1 | general disorders and administration site conditions | death | 261 | 7.12(6.25, 8.11) | 6.34(5.64, 7.13) | 1196.05 | 2.66(2.48) |
| 2 | general disorders and administration site conditions | drug ineffective | 119 | 2.77(2.3, 3.33) | 2.67(2.24, 3.19) | 126.53 | 1.41(1.15) |
| 3 | general disorders and administration site conditions | disease progression | 91 | 20.36(16.49, 25.13) | 19.5(16.03, 23.72) | 1594.34 | 4.28(3.98) |
| 4 | general disorders and administration site conditions | condition aggravated | 57 | 5.29(4.06, 6.88) | 5.17(4.01, 6.67) | 192.53 | 2.37(1.99) |
| 5 | general disorders and administration site conditions | asthenia | 51 | 4.01(3.04, 5.3) | 3.94(2.99, 5.18) | 112.36 | 1.98(1.58) |
| 6 | general disorders and administration site conditions | gait disturbance | 43 | 7.24(5.35, 9.8) | 7.11(5.3, 9.54) | 226.14 | 2.83(2.4) |
| 7 | general disorders and administration site conditions | therapeutic response unexpected | 28 | 22.87(15.73, 33.23) | 22.57(15.55, 32.75) | 574.94 | 4.49(3.96) |
| 8 | general disorders and administration site conditions | no adverse event | 20 | 3.73(2.4, 5.8) | 3.71(2.41, 5.71) | 39.6 | 1.89(1.27) |
| 9 | general disorders and administration site conditions | gait inability | 8 | 4.88(2.44, 9.78) | 4.87(2.45, 9.67) | 24.57 | 2.28(1.34) |
| 10 | general disorders and administration site conditions | catheter site pain | 3 | 44.53(14.28, 138.85) | 44.46(14.26, 138.57) | 126.37 | 5.46(4.04) |
| 11 | general disorders and administration site conditions | energy increased | 3 | 16.63(5.35, 51.7) | 16.61(5.33, 51.77) | 43.87 | 4.05(2.63) |
| 12 | nervous system disorders | amyotrophic lateral sclerosis | 79 | 1094.74(855.98, 1400.11) | 1052.55(831.95, 1331.65) | 68896.71 | 9.77(9.42) |
| 13 | nervous system disorders | aphasia | 36 | 38.38(27.57, 53.43) | 37.72(27.03, 52.64) | 1278.2 | 5.23(4.76) |
| 14 | nervous system disorders | speech disorder | 35 | 20.52(14.68, 28.68) | 20.19(14.47, 28.17) | 636.29 | 4.33(3.85) |
| 15 | nervous system disorders | cerebral infarction | 11 | 11.08(6.12, 20.06) | 11.03(6.13, 19.86) | 100.16 | 3.46(2.64) |
| 16 | nervous system disorders | dysstasia | 5 | 5.3(2.2, 12.75) | 5.29(2.19, 12.78) | 17.37 | 2.4(1.24) |
| 17 | nervous system disorders | dysgraphia | 4 | 17.77(6.65, 47.46) | 17.73(6.65, 47.24) | 62.95 | 4.14(2.87) |
| 18 | nervous system disorders | myasthenia gravis | 3 | 8.88(2.86, 27.58) | 8.87(2.85, 27.65) | 20.91 | 3.15(1.73) |
| 19 | nervous system disorders | muscle contractions involuntary | 3 | 26.41(8.49, 82.2) | 26.37(8.46, 82.19) | 72.86 | 4.71(3.29) |
| 20 | nervous system disorders | haemorrhagic cerebral infarction | 3 | 123.61(39.3, 388.79) | 123.43(39.6, 384.7) | 355.78 | 6.91(5.48) |
| 21 | respiratory, thoracic and mediastinal disorders | dyspnoea | 54 | 2.96(2.26, 3.88) | 2.91(2.26, 3.75) | 68.23 | 1.54(1.15) |
| 22 | respiratory, thoracic and mediastinal disorders | respiratory failure | 30 | 9.91(6.91, 14.22) | 9.78(6.87, 13.92) | 236.49 | 3.29(2.78) |
| 23 | respiratory, thoracic and mediastinal disorders | respiratory disorder | 7 | 7.05(3.36, 14.82) | 7.03(3.34, 14.81) | 36.2 | 2.81(1.81) |
| 24 | respiratory, thoracic and mediastinal disorders | choking | 4 | 6.48(2.43, 17.29) | 6.47(2.43, 17.24) | 18.47 | 2.69(1.42) |
| 25 | respiratory, thoracic and mediastinal disorders | asphyxia | 3 | 7.35(2.37, 22.84) | 7.34(2.35, 22.88) | 16.42 | 2.87(1.46) |
| 26 | respiratory, thoracic and mediastinal disorders | respiration abnormal | 3 | 10.08(3.25, 31.33) | 10.07(3.23, 31.39) | 24.47 | 3.33(1.91) |
| 27 | respiratory, thoracic and mediastinal disorders | dependence on respirator | 3 | 145.76(46.23, 459.58) | 145.55(45.79, 462.63) | 418.83 | 7.15(5.7) |
| 28 | musculoskeletal and connective tissue disorders | muscular weakness | 43 | 12.43(9.19, 16.82) | 12.19(9.08, 16.36) | 441.54 | 3.6(3.17) |
| 29 | musculoskeletal and connective tissue disorders | musculoskeletal stiffness | 9 | 3.48(1.81, 6.71) | 3.47(1.82, 6.63) | 15.86 | 1.8(0.9) |
| 30 | musculoskeletal and connective tissue disorders | muscle atrophy | 3 | 5.35(1.72, 16.62) | 5.35(1.72, 16.67) | 10.59 | 2.42(1) |
| 31 | infections and infestations | pneumonia aspiration | 13 | 9.63(5.58, 16.62) | 9.57(5.53, 16.57) | 99.7 | 3.26(2.5) |
| 32 | infections and infestations | device related infection | 6 | 11.13(4.99, 24.82) | 11.1(4.97, 24.79) | 55.03 | 3.47(2.4) |
| 33 | infections and infestations | injection site infection | 4 | 40.98(15.3, 109.71) | 40.9(15.35, 108.98) | 154.47 | 5.34(4.07) |
| 34 | vascular disorders | deep vein thrombosis | 7 | 3.81(1.82, 8.01) | 3.8(1.8, 8) | 14.47 | 1.93(0.92) |
| 35 | vascular disorders | poor venous access | 6 | 17(7.62, 37.94) | 16.95(7.59, 37.86) | 89.8 | 4.08(3.01) |
| 36 | injury, poisoning and procedural complications | fall | 30 | 2.79(1.95, 4) | 2.77(1.95, 3.94) | 33.96 | 1.47(0.95) |
| 37 | injury, poisoning and procedural complications | fracture | 4 | 6.34(2.37, 16.91) | 6.33(2.38, 16.87) | 17.92 | 2.66(1.39) |
| 38 | investigations | transaminases increased | 6 | 6.7(3, 14.94) | 6.68(2.99, 14.92) | 28.96 | 2.74(1.67) |
| 39 | hepatobiliary disorders | hepatic function abnormal | 8 | 4.7(2.35, 9.41) | 4.68(2.36, 9.29) | 23.17 | 2.23(1.28) |

ROR, reporting odds ratio; PRR, proportional reporting ratio; BCPNN, bayesian confidence propagation neural network; CI, confidence interval; 95%CI, 95% confidence interval; N, the number of reports;IC025, the lower limit of95% CI, for the IC.
